# Modulation of Human Sperm Mitochondrial Respiration Efficiency by Plant Polyphenols

**DOI:** 10.3390/antiox10020217

**Published:** 2021-02-02

**Authors:** Alessandra Ferramosca, Stefano Lorenzetti, Mariangela Di Giacomo, Paola Lunetti, Francesco Murrieri, Loredana Capobianco, Vincenza Dolce, Lamberto Coppola, Vincenzo Zara

**Affiliations:** 1 Department of Biological and Environmental Sciences and Technologies, University of Salento, I-73100 Lecce, Italy; digiacomomariangela@hotmail.it (M.D.G.); paola.lunetti@unisalento.it (P.L.); loredana.capobianco@unisalento.it (L.C.); vincenzo.zara@unisalento.it (V.Z.); 2 Department of Food Safety, Nutrition and Veterinary Public Health, Istituto Superiore di Sanità, I-00161 Rome, Italy; stefano.lorenzetti@iss.it; 3 Biological Medical Center “Tecnomed”, I-73048 Nardò (LE), Italy; francescomurrieri@centrotecnomed.it (F.M.); profcoppola@centrotecnomed.it (L.C.); 4 Department of Pharmacy, Health, and Nutritional Sciences, University of Calabria, 87036 Arcavacata di Rende (Cosenza), Italy; vincenza.dolce@unical.it

**Keywords:** quercetin, naringenin, luteolin, apigenin, genistein, resveratrol, sperm mitochondria, sex steroids

## Abstract

Plant bioactives, such as polyphenols, can differentially affect (positively or negatively) sperm quality, depending on their concentration. These molecules have been proposed as natural scavengers of reactive oxygen species (ROS) for male infertility treatment. However, few data are available about their effects on the molecular mechanisms related to sperm quality and, in particular, to sperm mitochondrial function. We investigated the effects of quercetin, naringenin, genistein, apigenin, luteolin, and resveratrol at the concentration of 0.1–1000 nM on mitochondrial respiration efficiency. Upon chemical exposure, spermatozoa were swollen in a hypotonic solution and used for polarographic assays of mitochondrial respiration. All tested compounds, except for apigenin, caused a significant increase in the mitochondrial respiration efficiency at the concentration of 0.1 nM, and a significant decrease starting from concentrations of 10 nM. The analysis of oxygen consumption rate in the active and in the resting state of mitochondrial respiration suggested different mechanisms by which the tested compounds modulate mitochondrial function. Therefore, by virtue of their ability to stimulate the respiration active state, quercetin, genistein, and luteolin were found to improve mitochondrial function in asthenozoospermic samples. Our results are relevant to the debate on the promises and perils of natural antioxidants in nutraceutical supplementation.

## 1. Introduction

Current evidence links oxidative stress to male subfertility and infertility [1]. Although adequate and controlled reactive oxygen species (ROS) levels play an important role in sperm physiology, high ROS levels negatively affect sperm quality and function [2,3,4,5]. Mature sperm cells are sensitive to reactive oxygen species’ damaging effects, because they lack proper repair machineries and have inadequate antioxidant capacity [6].

Different findings suggest a central role of sperm mitochondria in oxidative damage and related infertility [7,8], since these organelles, according to the “mitochondrial theory of aging”, are, at the same time, ROS generators and ROS targets [9,10,11]. Therefore, the biochemical mechanisms arranging mitochondrial metabolism and redox homeostasis are functionally linked.

Consequently, mitochondria have been identified as a potential therapeutic target and considerable effort has been made to evaluate the efficacy of natural compounds. In particular, many dietary natural polyphenols (mainly flavonoids) isolated from fruits, vegetables, and edible plants have been shown to modulate mitochondrial metabolism, organelle biogenesis, and redox status [12,13,14].

The protection of mitochondrial function by these plant bioactives may be important in explaining their beneficial effects on health. In this context, some studies [15] focused on the effects of the consumption of oral substances with antioxidant properties on sperm parameters, so that the use of biomolecules of plant origin for the improvement of male reproductive performance has become a modern trend in recent years.

For example, quercetin (QRC, 3,3′,4′,5,7-pentahydroxylflavone) is a mitochondria-targeted flavone [14,16] present in citrus fruits, berries, herbs and spices, tea, cocoa, red wine, and fruit juices that has been proposed as a natural scavenger of ROS in the treatment of male infertility. However, in the literature there are controversial reports highlighting the antioxidant as well as a pro-oxidant character of QRC, leaving much research to be carried out in this particular direction [17,18,19,20].

Similar to QRC, naringenin (NRG, 2,3-dihydro-5,7-dihydroxy-2-(4-hydroxyphenyl)-4*H*-1-benzopyran-4-one) is a natural flavonoid belonging to flavanones, commonly available in tomatoes, bergamot, and citrus fruits that merit further attention [21]. In particular, recent experiments carried out on boar semen [22] found that NRG was more effective against lipid peroxidation, while QRC acted as a stronger protective agent against protein oxidation.

Genistein (GEN, 5,7-Dihydroxy-3-(4-hydroxyphenyl)-4*H*-1-benzopyran-4-one) is a natural isoflavone compound present in soy products that seems to have positive effects only at certain concentrations on sperm characteristics, such as motility, viability, and mitochondrial activity [23].

Other molecules of plant origin have been studied for their effects on animal spermatozoa. Among them, the stilbene resveratrol (RESV, trans-3,5,4′-trihydroxystilbene) seems to protect the quality of the mitochondria, thus improving the sperm motility of bovine spermatozoa [24]. Another flavone, apigenin (API, 5,7-diidrossi-2-(4-idrossifenil)-4*H*-1-benzopiran-4-one), can ameliorate mitochondrial activity, antioxidant activities, and the intracellular ROS concentration of frozen-thawed boar spermatozoa [25]. Luteolin (LUT, 3′,4′,5,7-tetrahydroxyflavone) is also a common flavone that is abundantly present in various edible plants and seems to have protective effects against oxidative stress and mitochondrial dysfunction [26].

All these molecules have also been recognized to display estrogenic activity (Table 1) and to act as multi-functional endocrine disruptors, since they interfere with the enzymes needed for steroid biosynthesis and/or degradation [27]. Therefore, these compounds are also commonly known as phytoestrogens. In particular, QRC and GEN possess a weak estrogenic agonist activity [27,28]; LUT possesses a potent estrogenic agonist activity [27]; NRG has anti-estrogenic as well as estrogenic activities [29]; API has been reported to possess progestational activity or both progestational agonist and antagonist activity [27]; and RESV functions as a mixed estrogenic agonist/antagonist [30]. Indeed, androgen-like activities have also been suggested for all of them [31,32,33] (Table 1).

Interestingly, since sex steroid hormones are also implicated in controlling mitochondrial function, their hormone signaling is important for maintaining a proper mitochondria physiology, which is one of the major determinants of semen quality [34].

Therefore, we can conclude that natural antioxidants of plant origin differentially affect (positively or negatively) sperm quality, depending on their concentration. However, the information on the effect of various molecules on sperm quality is extensive, but, at the same time, fragmentary and/or partially inconsistent. This is mainly due to the very large variety of experimental conditions used in the studies carried out on this topic on gametes from different species.

The aim of this study is therefore to evaluate the possible effects of plant bioactives on sperm mitochondria, since these organelles play a key role in the modulation of sperm quality. In particular, we investigated the effects of QRC, NRG, GEN, API, LUT, and RESV on the mitochondrial respiration efficiency of human spermatozoa using the same experimental conditions. We took advantage of our already-established ex vivo assay of human sperm mitochondria [33,34], which were exposed to the tested molecules at the concentration range of 0.1–1000 nM. This range was considered because it broadly covers the estimated (nM range) dietary intake of these compounds.

The picture emerging from this investigation is relevant to the debate on the promises and perils of natural antioxidants of plant origin as nutraceutical supplements, suggesting caution in supplementation beyond levels attained in a healthy, plant-rich diet.

**Table 1:** Tested compounds and their hormone-like activities [27,28,29,30,31,32,33,35].

Molecule	Structure	Hormone-Like Activities					
		Estrogenic	Anti-Estrogenic	Progestational	Anti-Progestational	Androgenic	Anti-Androgenic
Quercetin (QRC)	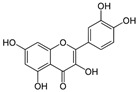	+			+		+
Naringenin (NRG)	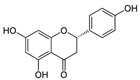	+	+				+
Genistein (GEN)	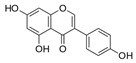	+	+				+
Apigenin (API)	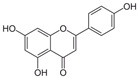	+	+	+	+		+
Luteolin (LUT)	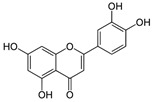	+			+		+
Resveratrol (RES)	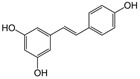	+	+			+	+

## 2. Materials and Methods

### 2.1. Tested Compounds

All chemicals were purchased from Sigma-Aldrich.

Nicotinamide adenine dinucleotide reduced (NADH, CAS no. 53-84-9) and carbonyl cyanide 4-chlorophenyl hydrazone (CCCP, CAS no. 555-60-2) were used as, respectively, positive and negative reference controls of the experimental model. Dimethyl sulfoxide (DMSO) was used as a blank control for each chemical treatment.

The plant bioactive chemicals used in this study (Table 1) were QRC (CAS no. 117-39-5), NRG (CAS no. 67604-48-2), GEN (CAS no. 466-72-0), API (CAS no. 520-36-5), LUT (CAS no. 491-70-3), and RESV (CAS no. 501-36-0).

The tested hormones included the human endogenous sex steroids testosterone (T, CAS no. 58-22-0) and 5α-dehydrotestosterone (DHT, CAS no. 521-18-6), 17β-estradiol (E2, CAS no. 50-28-2), and progesterone (P4, CAS no. 57-83-0) [33].

### 2.2. Sperm Samples

Sperm samples used in this study and respective spermiograms were provided by the biological medical center “Tecnomed” in Nardò (Lecce), Italy.

The research (new biochemical markers for the nanodiagnostics of male infertility) was approved by the Institutional Review Board of the Department of Biological and Environmental Sciences and Technologies at the University of Salento (10 November 2015) and was conducted in accordance with the Declaration of Helsinki. All experiments were performed in accordance with the relevant guidelines and regulations for research on human subjects. The donors (19–38 years old), who signed a written informed consent form for the use of their semen, did not have any conditions interfering with semen analysis (urogenital infections, leukocytospermia, or systemic diseases) and did not have a history of smoking, alcohol abuse or drug consumption; moreover, they did not receive antioxidant supplements or medication with a proven toxicity to fertility.

Semen samples were obtained by masturbation after 3–5 days of sexual abstinence and analyzed after liquefaction within 30 min, according to the World Health Organization (WHO)’s laboratory manual for the examination and processing of human semen [36]. Computer-assisted sperm analysis (CASA-SCA^®^: Sperm Class Analyzer, LabIVF Asia Pte Ltd., Singapore, Singapore) was carried out on all semen samples.

Sperm samples that satisfied the WHO guidelines’ criteria for normozoospermia (sperm total number >39 million; progressively motile spermatozoa >32%; morphologically normal spermatozoa >4%) were selected for this study. Sperm samples with a progressive motility of about 25% were also collected. Semen samples with similar characteristics (15 normozoospermic samples with a progressive motility from 32% to 40% and 10 asthenozoospermic samples with a progressive motility from 23% to 27%) were pooled. Sperm cells were collected by centrifugation at 800× *g* for 10 min at room temperature and resuspended in an isotonic salt solution [34] for mitochondrial respiration studies.

### 2.3. Human Sperm Exposure to Plant Bioactive Chemicals

10 × 10^6^ sperm cells/mL were incubated for 1 h at 37 °C with the chemicals reported in Table 1 at the concentrations of 0.1, 1, 10, 100, and 1000 nM. One hour (1 h) was chosen as incubation time, because an incubation of this duration allowed us to see the hormone-mediated effects on sperm mitochondria function [33].

A sperm control sample (blank) was incubated in the presence of 1% DMSO, which was used to dissolve the tested compounds.

Each experiment reported in this study included 6 separate assays for normozoospermic samples (blank, 0.1, 1, 10, 100, and 1000 nM of the tested chemical) or 2 separate assays for asthenozoospermic samples (blank and 0.1 nM).

### 2.4. Mitochondria Respiration Studies

After chemical treatment, spermatozoa were swollen in a hypotonic solution and used for polarographic assays of oxygen consumption [33,34].

Oxygen consumption was measured as nmol O_2_ × mL^−1^ × min^−1^⁄(10 × 10^6^ cells) by using a Clark-type oxygen probe (Hansatech oxygraph, King’s Lynn, UK) in the presence of a solution of respiratory substrates (10 mM pyruvate and 10 mM malate) and 0.76 μM of adenosine diphosphate (ADP).

V_3_ (the rate of oxygen uptake in the presence of pyruvate/malate and ADP) and V_4_ (the rate of oxygen uptake in the presence of pyruvate and malate alone) were measured and the respiratory control ratio (RCR) was calculated dividing V_3_ by V_4_ as an index of mitochondrial respiration efficiency.

### 2.5. Statistics

Each experiment was performed 4 times in the same experimental conditions. Data are expressed mean ± standard deviation. Student’s t-test was performed to detect significant differences between the control (blank) and chemically treated spermatozoa. Differences were considered statistically significant at *p* < 0.05.

## 3. Results

### 3.1. Effects of Plant Polyphenols on Human Sperm Mitochondria Respiration

To assess the reliability of our experimental model, NADH and CCCP were used, respectively, as positive and negative reference controls, since NADH is a substrate and CCCP is an uncoupler for mitochondrial oxidative phosphorylation [37].

**Figure 1:**
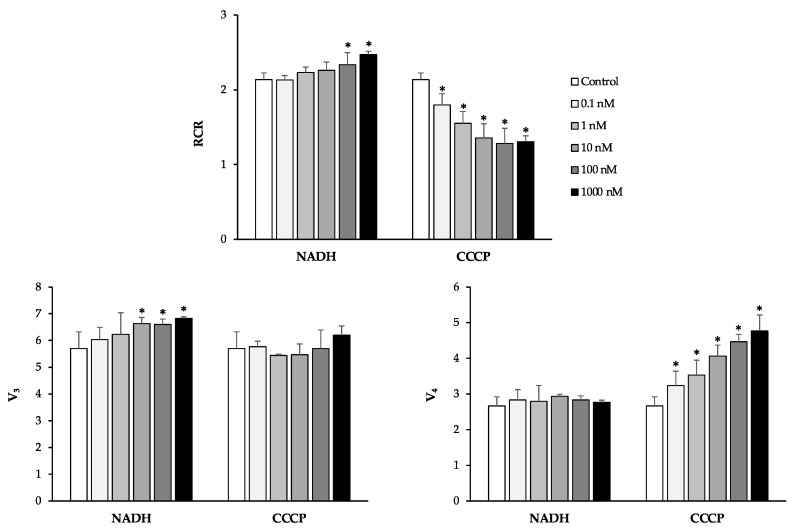
Experimental controls. Human spermatozoa were treated with the positive (nicotinamide adenine dinucleotide reduced, NADH) and negative (carbonyl cyanide 4-chlorophenyl hydrazone, CCCP) reference controls at the concentrations of 0.1–1000 nM. Oxygen consumption rates (V_3_ and V_4_) were measured and RCR (respiratory control ratio) was calculated as V_3_:V_4_ ratio. All data were subjected to Student’s *t*-test (* *p* < 0.05), *n* = 4.

As expected, NADH treatment caused a slight increase in the active state of mitochondrial respiration (V_3_) and an increase in the RCR values in a dose-dependent manner. Conversely, CCCP treatment caused a dose-dependent increase in the resting state of respiration (V_4_) and a dose-dependent decrease in RCR values (Figure 1).

**Figure 2:**
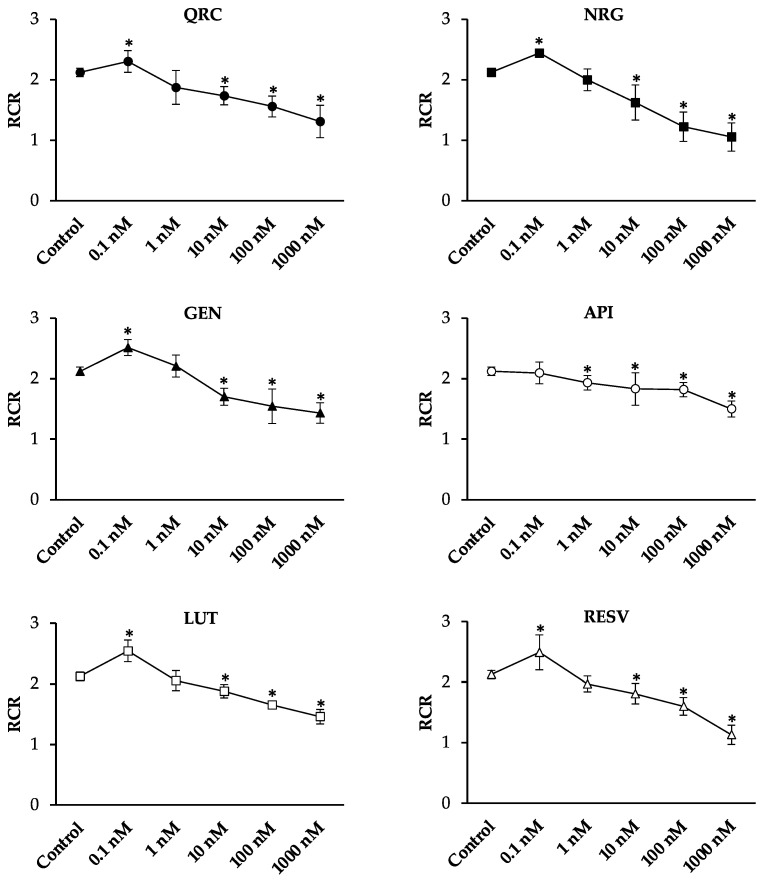
The effects of quercetin (QRC), naringenin (NRG), genistein (GEN), apigenin (API), luteolin (LUT), and resveratrol (RESV) on human sperm mitochondria respiratory efficiency. Human sperm cells were incubated with the chemicals at the concentrations of 0.1–1000 nM. The respiratory control ratio (RCR), which is an index of mitochondrial respiration efficiency, was calculated as V_3_:V_4_ ratio. All data were subjected to Student’s *t*-test (* *p* < 0.05), *n* = 4.

Then, sperm cells were treated with the plant polyphenols of interest, namely QRC, NRG, GEN, API, LUT, and RESV (Figure 2).

Except for API, we found a significant increase in RCR values when spermatozoa were exposed to the tested plant bioactives at the concentration of 0.1 nM. The increase was more remarkable for NRG, GEN, LUT, and RESV (about 20%) than for QRC (9%). Starting from concentrations of 10 nM, we found a significant decrease in sperm mitochondrial respiration efficiency, reaching the lowest value at the concentration of 1000 nM.

**Figure 3:**
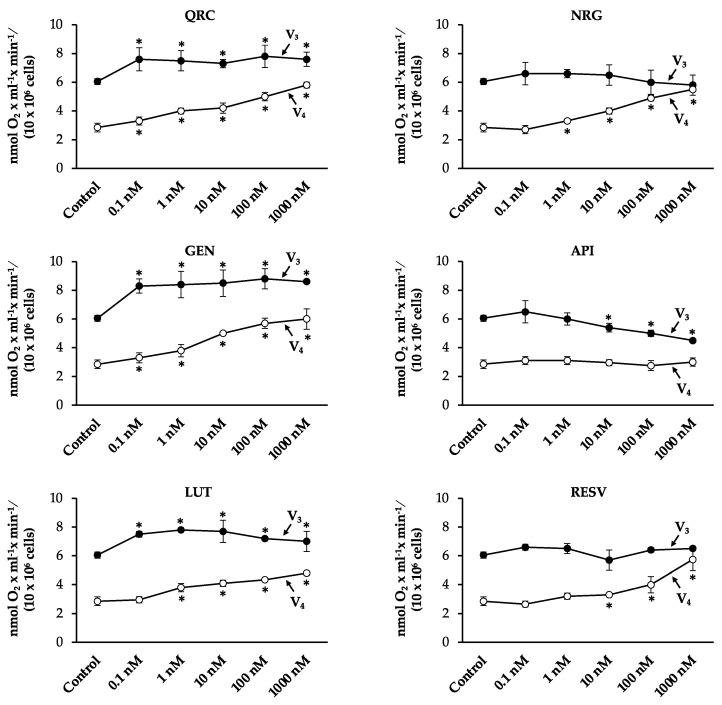
V_3_ and V_4_ values upon dose-dependent treatments with QRC, NRG, GEN, API, LUT, and RESV. V_3_ (the rate of oxygen uptake in the presence of pyruvate/malate and ADP) and V_4_ (the rate of oxygen uptake in the presence of pyruvate and malate alone) were measured as nmol O_2_ × mL^−1^ × min^−1^/(10 × 10^6^ cells). All data were subjected to Student’s *t*-test (* *p* < 0.05), *n* = 4.

The analysis of the V_3_ and V_4_ values (Figure 3) suggested different mechanisms by which the tested compounds modulate mitochondrial function.

In particular, QRC, GEN, and LUT stimulated the overall oxygen consumption. These molecules significantly increased V_3_ values at all concentrations, causing, at the same time, a significant increase in V_4_ values in a dose-dependent manner.

NRG and RESV did not affect the active state of mitochondrial respiration, but significantly stimulated the resting state of respiration, starting from a concentration of 1 nM and 10 nM, respectively.

API affected mitochondrial respiration efficiency in an opposite manner, causing a significant decrease in V_3_ values at the highest concentrations, without affecting V_4_ values.

**Figure 4:**
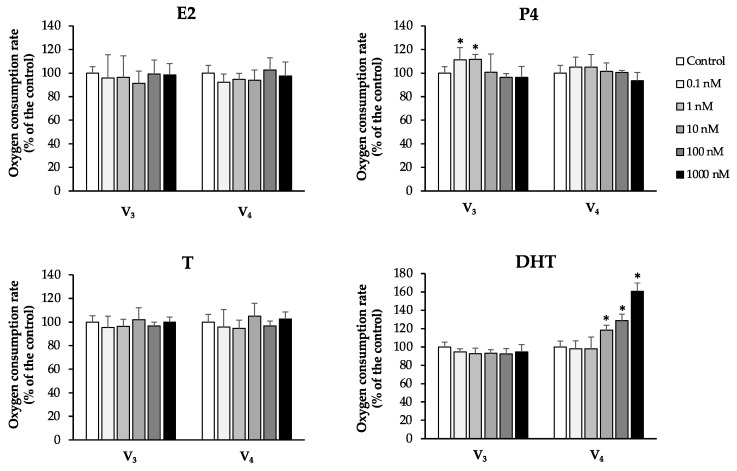
V_3_ and V_4_ values upon dose-dependent treatments with E2, P4, T, and DHT. The oxygen consumption rate obtained in the “blank” control was set to 100%. All data were subjected to Student’s *t*-test (* *p* < 0.05), *n* = 4.

All these molecules have also been recognized to possess hormone-like activities, in particular estrogen-like, progesterone-like, and androgen-like ones. Therefore, we compared the effects of the tested compounds on mitochondrial oxygen consumption with those obtained after sperm exposure to the endogenous estrogen E2, progesterone P4, and androgens T and DHT (Figure 4).

We found some similarities between the effects observed after NRG and RESV treatments and those observed after DHT exposure, since all these molecules stimulated the resting state of mitochondrial respiration at higher concentrations.

QRC, GEN, and LUT showed an intermediate behavior between that of P4 (the stimulation of V_3_ at the lowest concentrations) and DHT (the stimulation of V_4_ at the highest concentrations).

### 3.2. Effects of Plant Polyphenols on Mitochondria Respiration of Spermatozoa from Asthenozoosperic Subjects

The results reported in Figure 2 and Figure 3 suggest a positive effect of the tested plant bioactives QRC, NRG, GEN, LUT, and RESV (except API) only at the lowest concentration (0.1 nM).

Previous studies showed that spermatozoa with a progressive reduction in motility had dysfunctional mitochondria [38,39,40] and that the reduction in the respiratory efficiency was mainly associated with a decrease in the V_3_ values [37,38].

**Figure 5:**
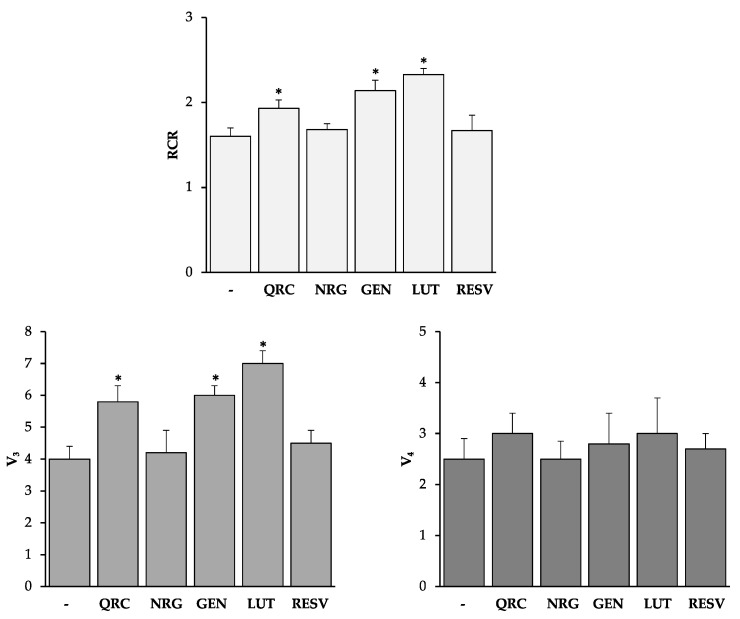
The effect of QRC, NRG, GEN, LUT, and RESV on the mitochondrial respiration of spermatozoa from asthenozoospermic subjects. Human spermatozoa were treated with QRC, NRG, GEN, LUT, and RESV at the concentration of 0.1 nM. The oxygen consumption in the active state of mitochondrial respiration (V_3_) and in the resting state (V_4_) of mitochondrial respiration was measured as nmol O_2_ × mL^−1^ × min^−1^/(10 × 10^6^ cells). RCR values were calculated as a V_3_:V_4_ ratio. All data were subjected to Student’s *t*-test (* *p* < 0.05), *n* = 4.

Therefore, we decided to test the effects of QRC, NRG, GEN, LUT, and RESV at the concentration of 0.1 nM on spermatozoa from asthenozoospermic subjects, with a motility of about 25%. We found that QRC, GEN, and LUT were able to improve mitochondrial function by significantly increasing V_3_ values (Figure 5).

In particular, RCR values were increased by 21%, 34%, and 46% and V_3_ values were increased by 45%, 50%, and 75%, after the treatment with QRC, GEN, and LUT, respectively.

## 4. Discussion

During the last decade, several studies have shown that some mitochondrial parameters, such as organelle integrity, respiratory activity, membrane potential, and ROS production, are strictly linked to sperm quality [41,42,43,44,45,46,47,48,49,50]. Additionally, poor sperm mitochondrial function is reported to be associated with seminal ROS levels. In fact, despite their key role in sperm energy metabolism to which glycolysis also contributes [51], mitochondria are highlighted as sources of pro-oxidative factors that are crucial in the alteration of oxidative homeostasis [7,14,42,52,53].

There is growing evidence that mitochondria are a powerful screening tool to assess the effects of several compounds, especially those with a hormone-like activity [54,55,56]. In this study we took advantage of our already established ex vivo human sperm mitochondria assay [33,34] in order to respond to the question: can mitochondria be a sensor for antioxidant compounds that might influence sperm function? This question arises from the fact that, in current medical practice, antioxidant therapy is widely used in the management of oxidative-stress-induced male infertility, although its action at the subcellular levels remains unclear.

Among many natural products, plant bioactives such as polyphenols have been extensively investigated for the treatment of male reproductive dysfunction and sperm quality decline [57]. At the same time, most of these molecules can target mitochondria to improve and/or restore their function by indirectly modulating the redox status, as observed in several diseases [14].

Therefore, in this study we tested the effects of some plant polyphenols (QRC, NRG, GEN, API, LUT, and RESV) at different concentrations (0.1–1000 nM) on human sperm mitochondria. This range was considered because it broadly covers the plasma concentration (nM range) of these compounds in Western populations. In fact, polyphenol plasma concentrations were estimated to be 0.01 μM (10 nM) in the European population, with a mean polyphenol intake of 0.5–0.8 mg/day. Concentrations were estimated to be 0.23 μM (230 nM) for vegetarians and vegans, with a mean polyphenol intake of 22.4 mg/day [58]. For example, the daily intake of QRC with a typical Western diet was estimated to range between 0 and 30 mg, with a median value of 10 mg. QRC is also available as a dietary supplement with a recommended daily dose of 200–1200 mg, or as a nutraceutical for functional foods within a concentration range of 10–125 mg/serving. Median maximum plasma concentrations of QRC (431 nM) were observed 360 min after the intake of 150 mg of quercetin [59].

At the tested concentrations, we observed a hormetic effect of polyphenols on human spermatozoa, because these molecules caused a significant increase in the RCR value, an index of mitochondrial respiration efficiency, at the concentration of 0.1 nM; starting from concentrations of 10 nM, mitochondrial respiration efficiency decreased.

Our results demonstrate that QRC was able to stimulate the active state of mitochondrial respiration at the concentrations of 0.1–1000 nM, causing, at the same time, an uncoupling between electron transport and ATP synthesis in a dose-dependent manner. This suggests that QRC exhibits both antioxidant and pro-oxidant activities on human sperm mitochondria, depending on its concentration.

These results are in agreement with the “QRC paradox in male reproductive dysfunction”, which can be explained by a biphasic concentration-dependent response of sperm cells (from a wide variety of species) to QRC [60,61,62,63,64,65], justifying the conflicting biological effects [66].

Molecular investigation of the mechanism by which QRC targets mitochondria suggested that, due to its chemical structure, QRC interacts directly with mitochondrial membranes and their components, affecting the production of ATP [67,68]. In particular, it has been proposed that QRC is able to competitively inhibit complex I at the Coenzyme Q-binding site, suppressing superoxide generation and allowing electron transfer to continue from NADH to complex III [68,69]. This evidence results in a more efficient coupling with ATP synthesis, which could justify the observed increase in RCR and V_3_ values. On the other hand, the dose-dependent increase in V_4_ values could be due to the interaction between flavonoids and lipid bilayers as well as with membrane proteins, which could influence the electric properties of mitochondrial membranes [70].

Moreover, the hydrophobic ring structure of QRC and the presence of hydroxyls as potential hydrogen bond donors prompted us to compare its activity to that of sex steroid hormones. Indeed, QRC has been shown to possess a P4 antagonist activity in both breast and endometrial cancer models [27]. In our experimental system, we found that QRC showed an intermediate behavior between that of P4 (the stimulation of the active state of respiration at the lowest concentrations) and DHT (the stimulation of the resting state of respiration at the highest concentrations). This is an intriguing aspect, since it could be supposed that sex steroid hormones and flavonoids can use similar mechanisms to target mitochondria.

Compared with QRC, GEN and LUT exhibited similar but more remarkable effects on human sperm mitochondrial respiration. Although there are very few studies on the effects of GEN and no studies on the effects of LUT on sperm quality [71,72,73], our results could be interpreted based on the proposed “mitochondriotropic” role of GEN [74] and of the protective effect of LUT against oxidative stress and mitochondrial dysfunction [26]. To our best knowledge, this is therefore the first evidence of a modulation of human sperm mitochondrial respiration by plant polyphenols, considered natural antioxidant compounds, in a dose-dependent manner.

Additionally, we found that GEN and LUT, along with QRC, were able to improve mitochondrial function in asthenozoospermic samples. This is an interesting aspect, because these results suggest the potential use of these molecules for sperm preparation to be used in assisted reproduction techniques (ART), especially in cases of asthenozoospermia. Our results are also in agreement with the improvement in human sperm motility observed after QRC treatment at appropriate concentrations [60].

In this context, it has also been suggested that the addition of NRG during semen storage may improve gamete quality [75,76]. However, in our experiments where NRG was added directly to sperm cells (without seminal plasma) we demonstrated that NRG significantly uncoupled mitochondrial oxidative phosphorylation starting from a concentration of 10 nM. Similar results were also obtained in endometriosis cells, where NRG has been shown to depolarize mitochondrial membrane potential [77].

Our data reveal some similarities between the effects observed after NRG and RESV treatment. Some studies reported that RESV acts as a regulator of male reproductive function [78,79] and it has been suggested that RESV arguably improves semen quality in humans [80] when this molecule is added to a cryopreservation medium.

The effects of RESV on mitochondria have been investigated in different experimental models, where it has been found that RESV induces both pro-oxidant and antioxidant effects on mitochondria [81]. Therefore, according to our results on sperm cells, high concentrations of RESV could be responsible for detrimental effects on mitochondria.

Finally, we found that, differently from the other tested plant polyphenols, API negatively affected mitochondrial respiration, causing a significant decrease in the active state of mitochondrial respiration in a dose-dependent manner. This result is in agreement with the hypothesis that API is an inhibitor of mitochondrial complex I [82].

Taken together, the results of the present study reveal that sperm mitochondria are a plausible main target of polyphenols, which can differently affect (positively or negatively) the organelle function, depending on their concentration. The modulation of sperm mitochondrial function could play a key role in the improvement of sperm quality.

We are aware of the limitations of this research, which represents a pilot and limited ex vivo experiment on the effect of single plant bioactives on sperm mitochondria function. However, we hope that our results could serve as a basis for future studies on antioxidant combinations in the improvement of sperm quality.

## 5. Conclusions

This study allowed for the clarification of the effects of selected plant polyphenols, considered natural antioxidants of plant origin, on human sperm mitochondria function. We found that all the tested plant polyphenols had a dose-dependent effect. In particular, QRC, NRG, GEN, API, LUT, and RESV caused a significant increase in the mitochondrial respiration efficiency at the concentration of 0.1 nM, and a significant decrease starting from concentrations of 10 nM.

Moreover, by virtue of their ability to stimulate the respiration active state, QRC, GEN, and LUT were found to improve mitochondrial function in asthenozoospermic samples.

Our results provide not only important insights for the debate on the promises and perils of natural antioxidants in nutraceutical supplementation, but also suggest that media containing plant-derived antioxidants could increase sperm quality. This is an intriguing matter, because the addition of plant bioactives at the appropriate concentrations could improve the outcome of ART programs, especially in cases of asthenozoospermia.

## Data Availability

Data is contained within the article.
